# Surgical Excision of Pulmonary Metastases of Dermatofibrosarcoma Protuberans

**DOI:** 10.7759/cureus.71076

**Published:** 2024-10-08

**Authors:** Adriana Nocera, Qianqian Zhang, Antonio Giulio Napolitano, Dania Nachira, Leonardo Petracca-Ciavarella, Maria Teresa Congedo, Alessandra Cancellieri, Giovanni Schinzari, Ketty Peris, Stefano Margaritora, Elisa Meacci

**Affiliations:** 1 Thoracic Surgery, Fondazione Policlinico Universitario “Agostino Gemelli” IRCCS, Università Cattolica del Sacro Cuore, Roma, ITA; 2 Anatomic Pathology, Fondazione Policlinico Universitario “Agostino Gemelli” IRCCS, Università Cattolica del Sacro Cuore, Roma, ITA; 3 Oncology, Fondazione Policlinico Universitario “Agostino Gemelli” IRCCS, Università Cattolica del Sacro Cuore, Rome, Italy, Roma, ITA; 4 Dermatology, Fondazione Policlinico Universitario “Agostino Gemelli” IRCCS, Università Cattolica del Sacro Cuore, Roma, ITA

**Keywords:** dermatofibrosarcoma protuberans (dfsp), fibrosarcomatous transformation, fs-dfsp, pulmonary metastases, uniportal-vats

## Abstract

Dermatofibrosarcoma protuberans (DFSP) is a rare malignancy of mesenchymal origin of medium-low grade with a tendency to local recurrences but not to distant metastases. We present the case of a 37-year-old male who underwent surgical resection of a 1.2 cm DFSP lesion on the left shoulder in May 2020. In the absence of a standardized follow-up protocol for DFSP, the attending physician opted for ultrasound monitoring every six months to detect any local recurrences. Due to chest pain and mild exertional dyspnea, a CT scan was performed three years post-excision revealed a 10 cm mass in the left lower lung lobe and two lesions in the right lung measuring 3.2 cm and 1.2 cm, respectively. Thoracotomy was performed in July 2023 to remove the large lesion in the left lower lobe, necessitating intrapericardial resection of the left lower pulmonary vein and extra-pleural dissection due to parietal pleural infiltration. Uniportal-video-assisted thoracoscopic surgery (U-VATS) was performed one month later to resect the lesions on the right side. Pathological examination showed high mitotic activity, cellularity, and nuclear pleomorphism, in the absence of other malignant mesenchymal neoplasms, suggesting fibrosarcomatous transformation of DFSP in the metastatic lesions (FS-DFSP). The patient remains disease-free under close radiological and clinical surveillance. Given the potential for aggressive transformation and the risk of recurrence and distant metastasis, our experience suggests including chest CT scans in the follow-up algorithms for DFSP.

## Introduction

Dermatofibrosarcoma protuberans (DFSP) is a rare cutaneous fibrohistiocytic neoplasm, accounting for approximately 1% of all soft tissue sarcomas. Although DFSP is characterized by slow growth and a low metastatic potential (less than 5%), it is known for its aggressive tissue infiltration, leading to a high rate of local recurrence. [1] In some cases, DFSP can undergo fibrosarcomatous transformation, a change that significantly increases the risk of metastasis and worsens the overall prognosis. These features make DFSP a complex and insidious condition, requiring careful clinical management. We present a complex case in which DFSP underwent fibrosarcomatous transformation, resulting in pulmonary metastases. The management of this patient necessitated the involvement of a dedicated multidisciplinary team due to the complexity and severity of the clinical scenario. This case underscores the importance of an integrated and coordinated approach in managing rare and aggressive neoplasms such as transformed DFSP.

## Case presentation

**Figure 1:**
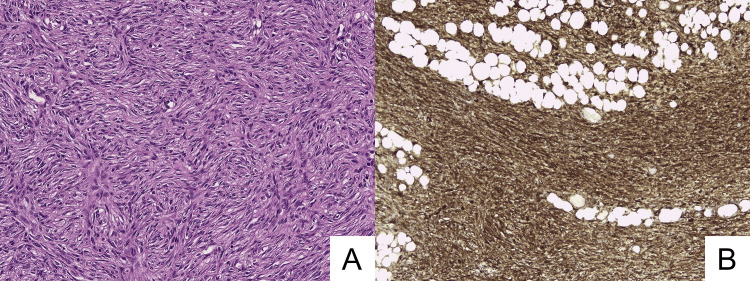
Primary shoulder lesion (DFSP) A) Hematoxylin-eosin staining: neoplastic cells are organized in storiform bundles. B) Immunohistochemistry: CD34+, fat infiltration.

In 2020, a 37-year-old male presented with a nodular subcutaneous formation on his left shoulder, which had been painful and rapidly growing for approximately a year. Since dermatofibrosarcoma protuberans (DFSP) was strongly suspected, the patient underwent surgical excision. Microscopic examination revealed a proliferation of monomorphic spindle cells, organized in storiform bundles (Figure 1A). The infiltrative nature was evidenced by subcutaneous fat trapping, resembling a honeycomb aspect. There was no significant mitotic activity and no necrotic foci. The neoplastic cells were CD34 positive (Figure 1B).

**Figure 2:**
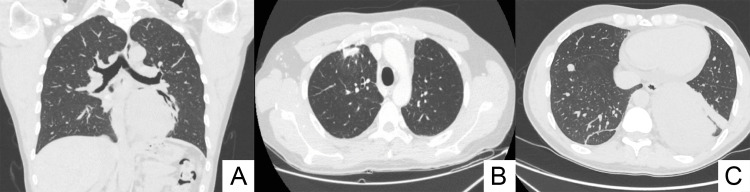
CT lung metastasis A) Lower pulmonary lobe completely occupied by mediobasal expansive formation,10x8cm, solid, inhomogeneous, extended in subpleural location, with signs of contiguity/compression in cranial location with the left atrium, at the confluence of the inferior pulmonary vein, the latter infiltrated and poorly recognizable in the proximal-medial section. B, C) Two solid, noncalcific nodular lesions of a secondary nature are appreciable in the anterior segment of the right upper lobe and antero-basal segment of the ipsilateral lower lobe, with dimensions of approximately 15 mm subpleural and 11 mm in the periscissural location.

The diagnosis of DFSP was confirmed on the surgical specimen. The lesion was completely excised with a wide safety margin (greater than 2 cm). After surgery, staging total body CT was performed which did not reveal distant metastases or suspicious local lymphadenopathy. The patient enters ultrasound monitoring every six months to detect any local recurrences. Due to chest pain and mild exertional dyspnea, in 2023 a chest x-ray was performed which showed pulmonary opacity in the left basal field and for this reason the patient independently performed an MRI which detected a 10 cm mass in the lower lobe of the left lung. Subsequent total body CT confirmed the presence of an expansive formation in the left lung and two additional nodular lesions on the right lung, measuring approximately 15 mm (subpleural location) and 11 mm (periscissural location) (Figure 2).

**Figure 3:**
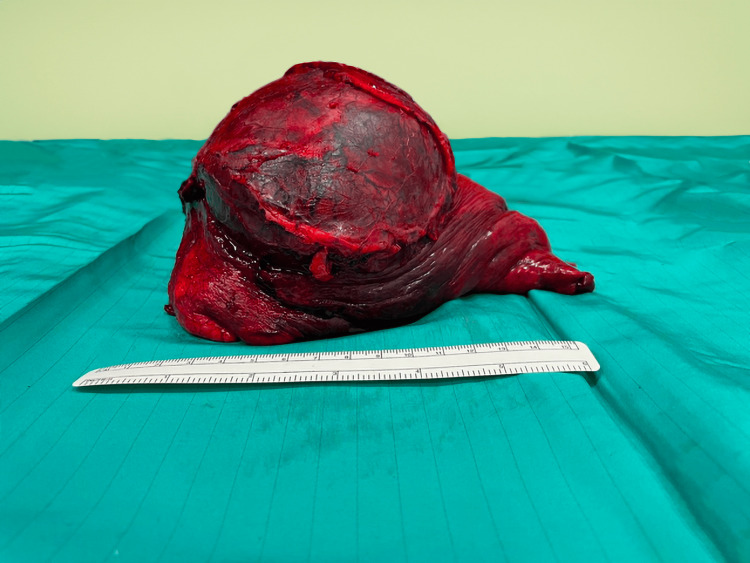
Left lower lobe: surgical specimen

The multidisciplinary team, strongly suspecting lung metastasis despite the low metastatic potential of DFSP, decided to proceed with surgical resection of lung lesions. Surgery to remove the mass occupying the left lower lobe was performed via left lateral thoracotomy at the fifth intercostal space. The neoplasm infiltrated dorsally into the parietal pleura, extending into the extrapleural space and periaortic mediastinal fat; therefore, the inferior pulmonary vein was prepared via an intrapericardial approach. The neoplasm is dissected via an extra-pleural route, however, it is not possible to obtain adequate control of the infiltrated peri-aortic plane and the costo-vertebral angle. It is therefore decided to perform a second mini service thoracotomy in the eighth intercostal space on the posterior acellar line through which the "en bloc" mobilization of the mass is completed. There were no post-operative surgical complications. Macroscopic examination of the surgical specimen revealed a 10 cm mass located 5 cm from the surgical margin (Figure 3).

**Figure 4:**
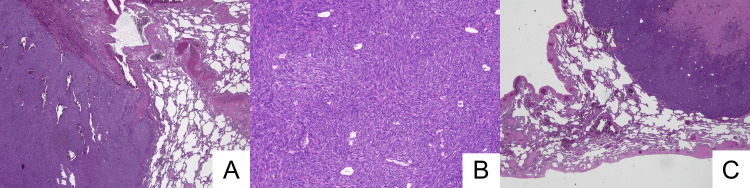
Histology of pulmonary localizations of fibrosarcoma A,B,C) Atypical proliferations of spindle cells arranged in fascicles often with herringbone appearance. Frequent mitoses (up to 30/10HPF) with presence of foci of necrosis.

Subsequently, surgical resection of lesions localized on the right upper and lower lobes of the lung was performed using a U-VATS surgical approach at the fifth intercostal space. Histological examination revealed an atypical proliferation of spindle cells, arranged in fascicular and herringbone patterns, with necrotic foci. The cellularity and mitotic activity were considerably higher (up to 30/HPF). Greater nuclear pleomorphism and hyperchromasia were observed (Figure 4).

The neoplastic cells were CD34 negative. The metastatic nature of all lung lesions was confirmed, and the diagnosis of metastatic fibrosarcoma (FS-DFSP) was made.

Until April 2024, radiological follow-up (FUP) with CT was negative for secondary effects or disease recurrence. In May 2024, due to disease recurrence, the patient is a candidate for Doxorubicin therapy without benefit. Genetic analysis using FoundationOne CDx sequencing did not demonstrate currently targetable mutations (mut. NF1). In July 2024 In light of the progression of the disease, it appears to be an indicator of a new line of therapy with Ifosfamide in a recovery regime. The patient is currently in good general condition. Performance status (PS) Eastern Cooperative Oncology Group (ECOG) scale score is 0 and the patient has no new onset symptoms.

## Discussion

Dermatofibrosarcoma protuberans (DFSP) is an uncommon, cutaneous fibrohistiocytic neoplasm that was first described by Darier and Ferrand in 1924. The term DFSP was coined by Hoffman in 1925 [1]. It accounts for 1% of all soft tissue sarcomas, with an incidence of 0.8 to 5 cases per million population each year. DFSP rates are highest among blacks, with a male-to-female ratio of 1:1 and a five-year relative survival rate of 99% [2-3]. Our patient is Caucasian.

Typically, DFSP presents as a painless plaque or mass, slow growing, and predominantly localized on the trunk (42%), upper and lower limbs (41%), and head and neck (16%). DFSP is a slow-growing tumor with low metastatic potential (<5%), but a high local recurrence rate, due to the aggressive tissue infiltrative attitude. The fibrosarcomatous transformation of DFSP may occur, leading to an increased metastatic potential and a less favorable prognosis [3].

The morphological features of DFSP are typically characteristic, but diagnosing DFSP from biopsy specimens can be challenging. Immunohistochemical features are helpful but not pathognomonic in diagnosis. Most DFSP (80%-100%) stain positive for CD34 and vimentin, while negative for factor XIIIA, desmin, smooth muscle actin, S100, and keratin. The positive stain for CD34 is also observed in some cases of cellular dermatofibroma (CDF), which is usually CD34 negative. In these cases, a biopsy specimen extending to subcutaneous tissue, which allows the evaluation of the eventual presence of an infiltrative pattern, is essential for a correct differential diagnosis.

A fusion between platelet-derived growth factor B-chain (PDGFB) and collagen type 1 alpha 1 (COL1A1) genes, caused by a reciprocal translocation t (17;22) (q22; q13), has been identified in 85-95% of DFSP [4]. Therefore, detection of the t (17;22) (q22; q13) translocation using polymerase chain reaction (PCR) or fluorescence in situ hybridization (FISH) staining, may be useful for confirming the diagnosis in ambiguous cases [4].

The fibrosarcomatous transformation is seen in 10-15% of DFSP [4]. FS-DFSP is a more aggressive neoplasm with a lower disease-free survival. On microscopic examination, the cellularity and mitotic activity are higher, the nuclear pleomorphism is more pronounced and necrosis foci can be present. Expression of CD34 is often decreased or lost in DFSP with fibrosarcomatous transformation. In our case, CD34 expression observed in the primitive cutaneous lesion is completely lost in lung metastasis.

Conventional cytotoxic chemotherapy has generally been ineffective for DFSP and currently plays a minimal role in treatment. For patients with DFSP with locally advanced or metastatic disease, tyrosine kinase inhibitors have produced response rates of 50% to 60% [5]. Therapies for metastases from fibrosarcomatous dermatofibrosarcoma protuberans (FS-DFSP) may include both medical and surgical treatments. Medical options include chemotherapy, immunotherapy, and targeted therapies such as the tyrosine kinase inhibitor imatinib, which has shown promising results [6]. Surgical treatments may include excision of metastases, but their effectiveness may vary based on the location and extent of the metastases.

In some cases, a combination of treatments may be necessary to manage metastases from FS-DFSP effectively. In any case, pulmonary metastasectomy is recommended if the metastases are fewer than five; if there are no extrapulmonary sites, it represents the gold standard. In the presented case, surgical excision of pulmonary metastases resulted in disease-free status during follow-up, indicating the efficacy of surgery in achieving local disease control. This highlights the importance of early detection and prompt surgical intervention in managing pulmonary metastases from FS-DFSP. However, this underscores the importance of follow-up not only locally but also in cases of low-grade malignancy sarcomas like in our patient. Currently, there are no studies regarding the effectiveness of radiological follow-up, but based on our experience, it would be advisable to undergo at least one chest CT scan every 12 months for the first five years. This is considering the propensity of such lesions to involve the lung parenchyma.

## Conclusions

The management of pulmonary metastases from dedifferentiated dermatofibrosarcoma protuberans (FS-DFSP) often presents a clinical challenge due to its rarity and potential aggressiveness. Surgical resection remains the cornerstone of treatment for localized and accessible lesions, offering a curative option with favorable long-term outcomes. Further studies are needed to elucidate optimal treatment strategies for pulmonary metastases from FS-DFSP, particularly in cases refractory to surgical intervention. Moreover, it would be advisable to consider implementing a structured follow-up protocol, especially for low-grade malignancy lesions, to prevent late diagnosis of metastases.
